# An agent-based model of social care provision during the early stages of Covid-19

**DOI:** 10.1038/s41598-022-20846-9

**Published:** 2022-10-03

**Authors:** Umberto Gostoli, Eric Silverman

**Affiliations:** MRC/CSO Social and Public Health Sciences Unit, University of Glasgow, Glasgow, G3 7HR UK

**Keywords:** Epidemiology, Preventive medicine, Computational science

## Abstract

Social care is a frequent topic in UK policy debates, with widespread concern that the country will be unable to face the challenges posed by the increase in demand for social care. While this is a societal problem whose dynamics depends on long-term trends, such as the increase of human lifespans and the drop of birth-rates, a short-term crisis, such as a pandemic, can affect the need and supply of social care to a considerable, although temporary, extent. Building on previous modelling effort of social care provision, we present an agent-based computational model to investigate social care provision in the context of a pandemic (using as an example, the early stages of the Covid-19 pandemic), and related mitigation policies, on social care demand and supply, using a proof-of-concept agent-based model (ABM). We show how policy solutions aimed at controlling the pandemic may have substantial effects on the level of unmet social care need and propose that such models may help policymakers to compare alternative containment policies, taking into account their side effects on the social care provision process.

## Introduction

Throughout much of the developed world, demographic trends, such as the increase of human lifespans and birth-rates drop, caused an increase in demand for social care, i.e., the provision of personal and medical care for people in need of assistance due to age, disability or other factors. In the UK, the social care supply is largely dependent on *informal* social care, or care provided free-of-charge by family members and loved ones, in order to meet the needs of the population. Informal care is enormously widespread in the UK and is much larger than the formal care infrastructure. The Family Resources Survey 2013/14 showed that there were 5.3 million informal carers in the UK^1^, while projections indicate that the number of people receiving informal care will increase by 60% in the period 2015–2035^2^. According to the Health Survey for England 2017, 68% of participants aged 65 and over reported receiving help from unpaid helpers, while 21% said they had received help from both unpaid helpers and paid helpers^3^.

While the dynamics of unmet social care need depends mainly on long-run demographic trends, such as the increase of human lifespans and the reduction of birth-rates over many decades, relatively short-term crises, such as global disease outbreaks, can significantly affect social care provision, both directly and indirectly, through the effect of the policies which health authorities implement to contain the pandemic. The Covid-19 pandemic has, indeed, increased the pressure on informal carers significantly. Carers UK estimates that before the pandemic, the UK had 9.1 million unpaid carers, and that the impact of Covid-19 on vulnerable people generated an additional 4.5 million unpaid carers, for a total of 13.6 million^4^. Out of those already providing care before the arrival of the coronavirus, 81% report spending more time on care than they did previously^5^. Carers UK also estimates that carers had provided £135 billion of care between March and November 2020, representing a substantial increase over previous years.

One important feature of this pandemic, which makes it a good example to investigate the interaction with social care provision, is its highly unequal effect across demographic groups: the virus has affected vulnerable populations of older people and adults with disabilities particularly strongly. In the United Kingdom, for example, Office for National Statistics data shows that 65% of deaths due to Covid-19 between 12 February 2021 and 6 August 2021 were recorded in adults aged over 75^6^. Amongst adults with disabilities, the risk of death due to Covid-19 was 3.1 times greater for more-disabled men as compared to non-disabled men, and 3.5 times greater for more-disabled women^7^. Given the greatly increased risk of death due to Covid-19 among the groups which have the highest levels of social care need, it is particularly important for policy makers to understand the interaction between the pandemic dynamics and social care provision, so that, when assessing the advantages and disadvantages of alternative containment policies, their *overall* effect, i.e., including the effect on social care provision, is taken into account.

In this paper, we investigate the interaction between the social care provision process and pandemic dynamics, using the example of the SARS-CoV-2 pandemic, commonly known as Covid-19. As such, this paper fills a gap between the (sparse) literature focusing on the modelling of social care provision and the growing literature focusing on agent-based models of pandemic dynamics. In this paper we present an agent-based simulation model of social care in the United Kingdom, which includes a model of the early stages of the Covid-19 pandemic and its impact on individuals. The novelty of our framework is represented by the integration of the pandemic dynamics with the process of informal social care provision, a framework through which we can therefore account for the interdependence between the two processes.

To be sure, the model we presented is not meant to provide specific policy recommendations, as they would require an evaluation of the desirability or value of various outcomes which can be only the result of a political process. Rather, we propose this framework as a tool allowing the policymakers to better assess the effects of the pandemic’s containment policies on social care provided. Moreover, while the demographic and institutional elements of the model presented in this paper have been developed to reflect the specific reality of the UK, the same framework can be straightforwardly used for other countries, by using the related country-specific demographic data and institutional settings.

### Previous works

Our model is composed of two main modules: a *social care provision* module and a *Covid-19 spread* module. The social care provision module adopted in this paper is the latest development of a modelling effort spanning the last decade^8–11^, whose main features will be described in the next section.

The adoption of the agent-based methodology to model informal social care provision is quite recent and previous literature in this field is very sparse. One study related to the social care model used in this paper is a mixed micro-simulation/agent-based care supply and demand model, called DemoCare, recently proposed by Spijker et al^12^. This model takes into account fertility, mortality and marriage rates for cohorts born between 1908 and 1968 to estimate the amount of care available from partners and children for people aged 50 or over. The DemoCare model uses micro-simulation to generate kinship networks of 10,000 representative agents, based on the demographic characteristics of each cohort, limiting the network to spouse, children, children-in-law and grandchildren. Then, through ABM simulations, they estimate the demand for care of these representative agents and the amount of this need which can be satisfied by spouse and children, taking into account whether they are working, their state of health and the needs for care of the rest of the family (e.g child care needs).

The social care provision model we adopt in this paper is fully agent-based, unlike DemoCare, and this methodology allows us to account for the interactions *between* kinship networks (which occur when agents belong to more than one ego network). This interaction which is particularly important when enlarging the group of care suppliers beyond partners, children, children-in-law and grand-children to include brothers, nephews, aunts and uncles. Other important differences, such as a more extensive role for *social status* (which in the DemoCare model is represented by agents’ educational levels) and the endogenous determination of *formal care* though the working agents’ care-work choice, will be discussed in detail in the next section.

Agent-based modelling methodology has only recently been applied widely to the study of pandemics. The module presented in this paper shares some features with the work of Wilder et al.^13^, who proposed an agent-based model of Covid-19 spread taking into account age and comorbidity distributions, age-stratified contact patterns and household structures. The model follows the standard SEIR agent classification, with four severity levels for infectious agents (asymptomatic, mild, severe and critical) and levels of infectiousness depending on the severity level (asymptomatic and symptomatic) and the stage of infection (before and after the onset of symptoms). In addition, agents are isolated if they are in the severe and critical severity levels while agents with mild symptoms become isolated after a number of days determined through a stochastic process. Agents can become exposed through in-household and out-of-household contacts, with the in-household contacts being more likely to transmit the virus. The out-of-household contacts are based on a country-specific contact matrix containing the mean number of daily contacts agents of an age group have with agents from each of the other age groups.

The model of Covid-19 spread we present in this paper, it the first attempt, to the best of our knowledge, to integrate social care provision with the pandemic’s dynamics (although limited to the first stages of it). As such, it includes significant differences compared to the above mentioned works. While the investigation of social care provision within the context of a pandemic represents certainly an addition to the literature on social care, our model contains important novel elements also with regards to Covid-19 modelling effort of Wilder et al. First, because of the integration with a social care provision model, our pandemic model has an additional exposure setting, represented by the social care-related interaction between agents. Second, in our model the agents have additional attributes such as their homes’ geographical location and the social status. These attributes have an important role in the generation of the contact networks (as we assume that agents close in location and social status are more likely to be part of the same contact network). Moreover, the agents’ social status affects the probability they will develop conditions of different severity levels (according to the epidemiological phenomenon of the social gradient in health). Finally, our model includes a behavioural module representing the decision process determining the agents’ degree of isolation, as a response to the various kinds of risks associated with Covid-19.

## Results

In this section, we present the simulations’ results with a particular emphasis on the inequalities characterising both the pandemic and the social care outcomes.

**Figure 1:**
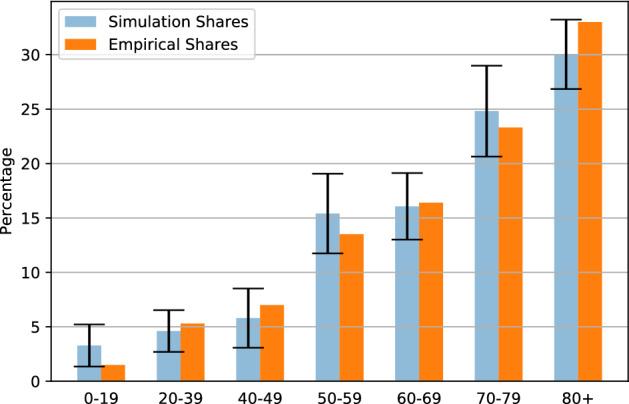
Share of hospitalized by age group.

The following graphs show, for each output, the mean across 20 repetitions, with 95% confidence intervals. Figure 1 demonstrates the model’s capacity to approximate the distribution of people hospitalized by age group. We can see that, under a lockdown policy which allows freedom of movement for social care purposes (which is, among the policies we considered in these simulations, the one which most resemble the policy adopted by the UK government), the empirical shares of hospital admissions by age, as reported in^14^, are within the 95% confidence interval for all the age groups, except for the 0-19 age group, which appears to be at the lower limit of the confidence interval (note, however, that there is a slight discrepancy between the grouping of^14^, where the first age group is the 0–17 group, and the grouping of the simulations’ outcomes, where the first age group is the 0–19 group. If we consider that the hospitalization rate increases with age, the empirical shares are higher for the 0–19 group and lower for the 20-39 group than they appear in Fig. 1).

Empirical studies have shown that, among non-pharmaceutical interventions, lockdowns have been the most effective ones in reducing the spread of Covid-19^15,16^. In the next set of figures, we compare the effects of two lockdown policies to the benchmark ‘no-lockdown’ scenario. In these figures, *Policy 1* refers to the partial lockdown policy, i.e. a lockdown which allows movement for social care purposes, while *Policy 2* refers to the ‘full lockdown’ policy. In both cases, the lockdown is imposed 3 days after the first death, and is lifted after 90 days.

**Figure 2:**
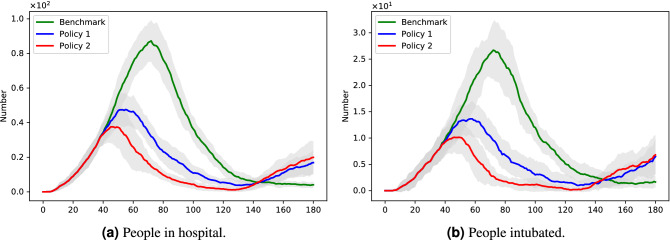
Policy comparison: outcome dynamics.

**Figure 3:**
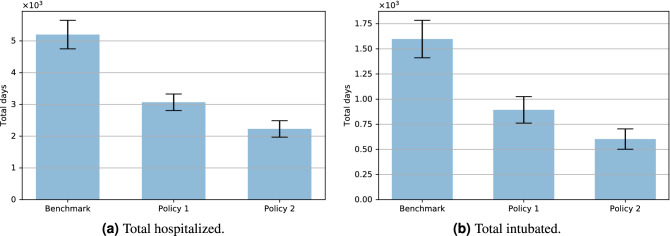
Policy comparison: aggregate outcomes.

Regarding the effects of these policies on the pandemic, we can see from Fig. 2a and b that the two policies reduce the height of the peak significantly; even a partial lockdown reduces the maximum number of people in hospital and on the ventilator by half. Figure 3a and b confirm this positive effect, by showing the *total* number of days of hospitalization and intensive care. We can see that Policy 1 and Policy 2 reduce the number of days of hospitalization by 40% and 60% respectively compared to the benchmark, and the percentage reduction of the number of days of intensive care is higher still.

**Figure 4:**
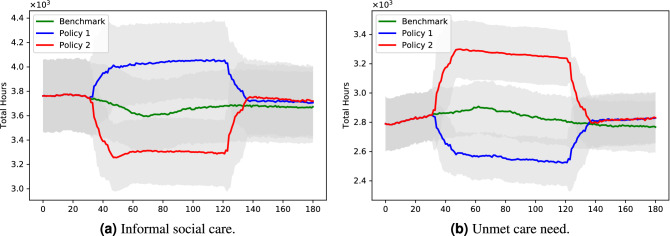
Policy comparison: care dynamics.

The last four graphs show the effect of these two policies on social care provision. Figure 4a shows that the two policies have opposite effects on the amount of informal care provided: while total lockdown reduced the amount of informal care (as potential suppliers cannot provide care to people in need living in other households), partial lockdown increases the informal care provided, as under partial lockdown people have more time to allocate to social care (an effect which is consistent with empirical observations). The effect of this variations of care supply on unmet care need are shown in Fig. 4b, where we can see an increase of unmet care need under Policy 1 and a decrease of it under Policy 2.

**Figure 5:**
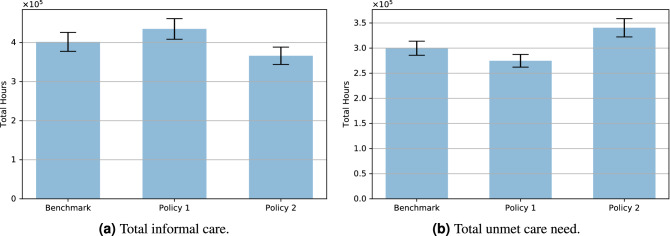
Policy comparison: care aggregate outcomes.

The following two figures show the differences between policies in terms of care provision and unmet care. From Fig. 5a and b we can see that the two policies have opposite effects on the amount of informal care and, therefore, unmet care need. While Policy 1 has a positive effect with regards to social care provision and unmet care need, the opposite is true for Policy 2, with the level of unmet care need with this latter policy being more than 20% higher than the level of unmet care need under the former policy.

To sum up, we can say that our simulations show that while Policy 1 has a positive effect on hospitalisations, intubations and unmet care need, in the case of Policy 2 the policy maker faces a trade-off posed by the even greater positive effect on the pandemic outcomes and an increase of about 13% of the level of unmet care need.

## Sensitivity analysis

Typically, the results of ABM simulations depend on many parameters which are characterized by a high degree on uncertainty. In our model, in particular, there are parameters related to the agents’ behaviour and the social networks which are not empirically determined and for which we must perform a sensitivity analysis in order to determine their importance in driving the simulations’ outcomes. In this sensitivity analysis, we chose the number of hospitalisations as the main output of interest.

Table 1 shows the total effect on the outcome’ variance for the eight parameters we included in the sensitivity analysis, the first four of which determine the agents’ behaviour, while the last four affect the size and structure of the social networks. To determine the ranges, we halved (lower bound) and doubled (higher bound) the parameters’ benchmark values.

**Table 1:** Sensitivity analysis.

Parameter	Behaviour affected	Range	Total effect
$\phi$	Domestic/care prudence	[0.25, 1.0]	2.0
$\xi$	Other-regarding concern	[0.005, 0.02]	33.04
$\omega$	‘Income-effect’	[0.25, 1.0]	2.28
$\mu$	‘Base’ sensitivity to outcomes	[20, 80]	27.06
*s*	Number of contacts	[0.05, 0.2]	35.54
*r*	Same-class contacts	[0.1, 0.4]	0.68
*h*	Same-area contacts	[0.001, 0.004]	1.05
*k*	Same-friends contacts	[0.05, 0.2]	2.81

We can see that the ‘base’ sensitivity to risk factors ($\mu$) and the effect of income ($\omega$) on the overall sensitivity are two of the most important parameters, with an effect on the overall variance of total hospitalizations of 35% and 27% respectively. The effect of the knowledge of infection on the behaviour within the household and during the care provision ($\phi$) is the second most important parameter, determining about 33% of the total variance of the outcome. The other-regarding preferences (affecting the behaviour of knowingly infected people) and the parameters affecting social network size and structure appear to have a marginal role in driving the outcome we considered.

## Discussion

In this work, we presented a ‘proof of concept’ of an agent-based framework integrating a model of social care provision with a model of Covid-19 spread, during the early stages of the pandemic. This novel framework, allowed us to study the effect on the provision of social care of both the Covid-19 spread and the public health measures taken to contain it. We showed that the model could be used to evaluate the relative advantages and disadvantages of alternative policies, and can therefore represent an important decision-support tool for policy-making decisions in this complex and highly uncertain context.

The model results demonstrate that policy-makers have a trade-off to consider when imposing public health restrictions in these circumstances. When lighter restrictions are imposed, informal social care provision increases, as informal carers have more time available and are not restricted from providing care; however, under these conditions the negative outcomes of the pandemic are more modestly reduced. Conversely, under more severe restrictions informal social care between households becomes impossible, worsening care outcomes through a significant increase in unmet care need, while the effects of the pandemic are more effectively contained. Models like this one could be used to investigate the nature and extent of these public health trade-offs, so that policy-makers may make informed decisions based on the current state of the pandemic.

The results also reflect the social gradients of health and care present in these scenarios. Unmet social care need is distributed highly unequally, with the lowest income quintile having four times as much unmet care need as the highest quintile. The sensitivity analysis shows that the number of contacts is a significant factor driving these results, which suggests that the higher income quintiles benefit from having jobs with greater inherent flexibility, which enables them to work from home in larger numbers and take more time off work to provide informal care.

Being this work a proof-of-concept, the framework presented in this paper contains many simplifications as our purpose was not to provide a complete model to make accurate forecasts but rather to develop a minimal model focusing on the interdependence between the pandemic dynamics and the informal social care provision process. Therefore, we included a ‘minimal’ model of the Covid-19 spread, which we aim to extend in future works. For this reason, We did not include, at this stage, important factors affecting the pandemic dynamics, such as the environmental and the institutional context, and we restricted our analysis to the stage of the pandemic *prior* to the introduction of vaccines. In addition, we did not include the effects of common interventions such as face coverings. In our future work we will continue to refine and extend this model to maximise its utility for public health policy-makers.

## Methods

This computational modelling study was carried out following all relevant guidelines; note that we only use publicly available population data, such as the Human Mortality Database^17^, Eurostat^18^ and the Office for National Statistics^19^. No human experiments were performed, nor was any human data collected during the course of this study.

The model is composed of three main modules:

- A demographic module (from year 1860 to year 2019, with yearly steps).
- A social care module (from year 2020, for 180 daily steps).
- A Covid-19 spread module (from year 2020, for 180 daily steps).

First, the *demographic module* creates a population on the UK with a realistic demographic structure, starting in the year 1860 and running until the year 2020 in one-year time steps. Then, from the beginning of the year 2020, a *social care module* and a *Covid-19 spread module* generate the social care provision process and the epidemiological progression of the pandemic respectively. These process then proceed for 180 one-day time steps (i.e., we simulate the first 6 months of the pandemic).

### The demographic module

The initial population of couples is randomly distributed on a 812-cell grid approximating the geography of the United Kingdom. Agents live in houses which form towns, with the density of those houses varying in rough proportion to UK population density. The agent population is scaled down from real UK levels at a factor of roughly 1:10,000. The simulation begins in the year 1860, which allows sufficient time for the population dynamics to stabilise before 1951, at which point UK Census mortality and fertility data is incorporated into the simulation^17,19^. Agents form partnerships, have children, start working (and earn an income), relocate, retire and die, according to sub-modules the details of which have been described in previous works^8–11^ and a summary of which is reported in the Supplementary Information.

Here we only mention the socioeconomic structure of the population generated by the demographic module: agents belong to one of five socioeconomic status groups (SES groups), based on the Approximated Social Grade from the Office for National Statistics, redistributed as in Gostoli and Silverman^10^. Moreover, the model contains a *social mobility* process: an agent is assigned the SES group associated with the education level he has reached, with a probability of moving further up the education ladder depending on the household’ income and the parents’ level of education. The introduction of SES groups has a number of effects on the various stages of agent life-courses: a higher SES is associated with lower mortality and fertility rates; higher hourly salaries; and lower salary growth rate. Socioeconomic status affects the agents’ wealth, which is randomly assigned to agents according to their accumulated salaries to reproduce the 2016 UK wealth distribution. The socioeconomic position of an agent affects social care supply within the household through the agent’s income, as we assume that the share of income allocated to care supply increases with the household’s per capita income. Moreover, the inclusion of agents’ SES allows us to take into account the *social gradient* in health, which affects both the social care demand, with the probability of transition to higher levels of care need being higher for agents of lower SESs, and the pandemic dynamics, with the probability of developing more severe infection courses depending negatively on the agents’ income level.

### The social care module

The social care module simulates the social and child care provision processes, starting from the year 2020, for 180 days. This module is based upon previous work on simulating child care provision^11^. The social care module can be conveniently described by looking at three key aspects:

- The care demand.
- The care supply.
- The care provision process.

#### Care demand

In the model, total care demand is given by two components: *childcare* and *social* care demand. As for the latter, agents begin the simulation in a state of good health, and subsequently may develop care needs according to age-, gender- and SES-specific probabilities. Table 2 shows the five possible categories of care need, and the hours of care need required at each level. In line with the current understanding of care need progression, we assume that agents who develop a condition requiring care do not recover, but instead progress to higher levels of severity and care need over time. This progression through the care need levels depends on age, gender, SES, and the cumulative unmet care need experienced by that agent. We therefore assume that large amounts of unmet care need increase frailty.

**Table 2:** Care need categories/levels and number of hours of care required.

Care need category	Care need level	Weekly hours of care required
None	0	0
Low	1	8
Moderate	2	16
Substantial	3	36
Critical	4	84

As for childcare need, we assume that all children, with the exception of newborns, have identical childcare need, requiring 10 hours of care each day. The net care need for each child agent varies by age, given the presence of child care and education policies targeted at specific age groups. Newborns have a much higher care need which must be provided by their mother, who in turn allocates all her care supply for the newborn.

#### Care supply

In this model, the care is provided through three main sources: informal care, privately paid-for formal care and government care. As for informal care, it is provided by the agents’ network of relatives. Agents requiring care have kinship networks linking them to households with members having a consanguineous or affinal relationship with that agent. Within these networks we define ‘degrees’ of kinship, based on the distances between the agent and each household in the network. This kinship distance ranges from 0 for agents in the same household, to III (uncles and aunts, or nieces and nephews). The supply of social care available to an agent is determined by the size of its kinship network, the kinship distances in that network, and the individual states of household members contained in that network. The hours of care supply that can be provided by each agent according to status and network distance are shown in Table 3. In addition to kinship distance, physical distance also impacts care provision. In this model we assume that care receivers may only receive informal care if the provider is in the same town. We also assume that kinship distance restricts the provision of formal care; private paid-for care occurs only between members of the same household, or between parents and children.

**Table 3:** Amount of care agents can provide depending on their status and kinship distance from the care receiver.

Agent status	Household (D 0)	D I	D II	D III
Teenager	12	0	0	0
Student	16	8	4	0
Employed*	16	12	8	4
Retired	56	28	16	8

*Employed agents can provide additional care if they choose to reduce their working hours (i.e. in case it is more convenient than using income to pay for formal care. See the Formal Care section for details).

Formal care may be provided within the care receiver’s household or by households with first-degree kinship relationships to the receiver. Income allocated for care may be used to buy private paid-for care, or to reduce hours spent at work in order to provide informal care (meaning that the income allocated in this case represents unearned income, rather than spent income). The care receiver may also purchase private care with their own wealth. The share of their wealth allocated to formal care is positively correlated to their overall financial wealth.

As outlined in previous work^11^, agents with care needs may be eligible for publicly funded care, via a government-funded care scheme based upon the framework in place in England (see the Supplementary Information for details).

#### Care provision process

In this model we adopt the care allocation process developed in our previous work^11^. We simulate care allocation as a negotiation taking place across agent kinship networks. We assume that childcare needs have priority over social care needs. Therefore, the allocation process unfolds in two stages: 1) the available care supply (available time/income for care) is allocated to child care need; 2) remaining resources are provided to social care needs (we refer the reader to the Supplementary Information for details of the care allocation process).

In both stages the allocation process randomly samples one care-receiving unit (which is an individual in the case of social care, or a household in the case of child care), with a probability proportional the unmet care need of that unit. The care receiver is then linked with a care-providing household in the receiver’s kinship network; potential care-giving households are sampled with a probability proportional to that household’s care supply. After the care supplier has been chosen, a 2-hour unit of care is provided from one member of that household with available supply to the care receiver. If the supplying household is at distance I, then that household may choose to provide either time (in the form of informal care) or income (in the form of formal care).

When choosing between providing assistance in the form of income or time, the choice is made depending on the hourly wage of the worker in the supplying household with the lowest wage. If the price of formal care is higher than that wage, then the supplying agent will prefer to take time off work to provide informal care; if the price of formal care is lower, then the agent will prefer to purchase that care and remain in work.

### The Covid-19 spread module

Building on the framework underlying our previous social care models, we introduce a model of Covid-19 spread which takes into account UK demographic structure, income distribution, age- and income-specific social mixing patterns, household structure and social care provision networks. The model does not include a range of environmental and institutional factors (e.g. weather, temperature, public health spending, among others) which have been found to affect the different pandemic dynamics between countries^20–22^. By modelling the pandemic dynamics in a single country (the UK), we can consider these contextual elements to be fixed, and we can focus on the socio-economic factors determining the inequalities of the Covid-19 outcomes within the UK.

As in the standard SEIR model, the agents can be in one of four states: *susceptible*, *exposed*, *infectious* or *removed* (i.e. recovered or deceased). As for the conditions of the infected agents after the virus incubation period, we distinguish between: *asymptomatic*, *mild symptomatic* (i.e., not hospitalized), *severe* (i.e., hospitalized but not in intensive care) and *critical* (i.e., in intensive care).

An infected agent is given a *viral load*, which is sampled from a standard uniform distribution. The viral load determines the agent’s *contagiousness* and, together with their age and income quintile, the severity of their symptoms (which we call the agent’s *symptoms severity index* ($\pi$)). The severity index affects both the agent’s mobility (which is equal to 0 for those hospitalized) and the probability that it takes a test to determine whether it is infected.

The main features characterizing our model of the pandemic are:

- The inclusion of a *behavioural module* determining the agents’ behavioural response to the risks of being infected or infect others.
- A multi-setting exposure process, composed of: a *social setting*, a *domestic setting*, and a *social care setting*.
- Age-, income- and gender-specific probabilities of developing different *virus infection courses*.

#### Behavioural module

While in standard SEIR models, the behavioural reaction is implicit in the probability of disease transmission, agent-based modeling allows us to develop an *explicit* model of the way the agents’ modify their behaviour in response to the pandemic’s spread. In this model, the agents react to the risks posed by the pandemic by reducing their social and work activities – by self-isolating. We assume that the agents can be concerned about two kinds of risk:

- The risk of being infected, if susceptible or if unknowingly infected (e.g. asymptomatic agents).
- The risk of infecting others, if knowingly infectious.

Infected agents become aware of their infection status if they take a test, which is taken with a probability that depends on the severity of their symptoms.

As for susceptible agents, who will be concerned about the risk of being infected, the factor by which they reduce their social and work activity (the *mobility reduction rate* (*m*)) depends on their sensitivity to the virus spread. The variable measuring the spread ($\rho$) is represented by the weighted moving average of the number of *new cases*, relative to the size of population. Formally, the mobility reduction rate is given by:

1 $$\begin{aligned} m_b = \frac{1}{e^{g\rho }} \end{aligned}$$

where *g* represents the susceptible agent’s overall *sensitivity* to the risk of being infected, which depends fundamentally on two parameters of the behavioural model:

- The *infection fatality rate* (*f*). It is a characteristic of the virus, and it is constant as during the simulation the virus does not evolve. For the agents, it represents the expected ‘cost’ of being infected and it depends on the agents’ age, income and gender.
- The ‘base’ sensitivity to infection’s outcomes ($\mu$). It is a behavioural parameter representing the ‘strength’ of the agents’ reaction to a unit increase of the infection fatality rate.

For a not-working agent (e.g., a retired agent), the degree of isolation does not affect its income and the occupational factor is not relevant, so the agent’s sensitivity to risk becomes:

2 $$\begin{aligned} g^{s}_u = f\mu \end{aligned}$$

In case the agent is working, their sensitivity to the risk of being infected depends also on:

- The agent’s *income* factor ($\omega$)
- The agent’s *occupation* factor ($\sigma$)

If the agent is working, indeed, reducing its mobility may mean reducing its working hours and therefore its income. Agents with a relatively high income can afford a higher reduction of their working hours compared to agents with a low income. Finally, agents with different kinds of occupations differ in their capacity to work from home. In line with empirical observations^23^, we assume that people of low social status have jobs with less inherent flexibility and are less able to work from home, compared to the jobs of people of high social status. The agent’s income quintile is used as a proxy for its social status. Formally, the agent’s sensitivity to the risk of being infected for a working agent is given by:

3 $$\begin{aligned} g^{s}_w = f\mu \omega \sigma \end{aligned}$$

where *g* is a proportionality parameter and $\omega$ is an increasing function of the agents’ income quintile.

For the reasons mentioned above, therefore, we should expect old people of high social status to be the most sensitive to the risk of being infected, and young people of low social status to be the least sensitive; in other words, we should expect agents of the former category to adopt a stricter isolation regime compared to the latter, for a given increase in case numbers.

As for the knowingly infectious agents, who will be concerned about the risk of infecting others, we assume that their mobility reduction depends on their sensitivity to the share of the susceptible population *S*:

4 $$\begin{aligned} m_b = \frac{1}{e^{g{S}}} \end{aligned}$$

For working agents, once again the sensitivity depends on the income and occupation, but differently from the susceptible agents, we assume that it depends on the *general* infection fatality rate, *F*, (as opposed to the agent-specific infection fatality rate) as it is the variable measuring the virus fatality among the population. Formally, the infectious agents’ sensitivity to the share of susceptible is given by:

5 $$\begin{aligned} g^{i}_w = \mu \xi {F}\omega \sigma \end{aligned}$$

and for unemployed agents:

6 $$\begin{aligned} g^{i}_u = \mu \xi {F} \end{aligned}$$

where $\xi$ is a parameter representing the ‘strength’ of the agents’ social (or other-regarding) preferences (i.e., how much they care about others’ well-being).

Apart from behavioural reactions to pandemic risks, the mobility of infected agents who are not asymptomatic may be reduced because of the debilitating effect of the virus. We assume that the extent to which the infected agent’s mobility is reduced depends on its symptoms severity index, $\pi$, according to the formula:

7 $$\begin{aligned} m_v = (1-\pi )^\eta \end{aligned}$$

where $\eta$ is a parameter determining how the mobility decreases as the severity of the agent’s symptoms increase. Therefore, the overall agent’s mobility reduction rate *m*, will be given by:

8 $$\begin{aligned} m = min(m_b, m_v) \end{aligned}$$

#### Exposure settings

In this model, a susceptible agent can become exposed in three settings:

- Informal social care setting.
- Domestic setting.
- Social setting.

As for the *social care setting*, agents with social care needs interact with relatives providing care from other household, a process through which the virus spreads. We assume that the probability of being infected by the care receiver or the care supplier depends on the contagiousness of the infected agent, on the duration of the interaction and, if the person carrying the virus is the care receiver, on whether the person knows about their infection status. If the receiver knows they are infectious, we assume that both the care receiver and the care supplier adopt a prudent behaviour which reduces the risk of contagion by a certain factor $\phi$ which is a parameter of the model. When the care supplier is infectious and aware of their status, we assume instead that they do not provide any care supply.

With regards to *domestic interaction*, agents can be infected through interactions within their household. The capacity of an infected household member to transmit the virus to a susceptible agent depends on their contagiousness (their viral load) and whether the infected agent knows they are infected. If the agent knows they are infected, we assume that households adopt a prudent behaviour reducing the risk of contagion by a certain factor $\phi$ which is a parameter of the model.

Finally, agents engage in a series of *social interactions* through which they come into contact with agents which are part of their *ego network*. These ego network of an agent is characterized by a size (i.e. the total number of contacts) and a distribution of contacts by age group, which depend on the agent’s age group, according to empirical findings on social mixing patterns (^24–26^). In line with findings of empirical studies of the effect of social status on social networks^27^, we assume that the network size increases with the agents’ social status (represented by their income quintile), according to a factor *s* which is a parameter of the model. The probability that two agents are part of the same network depends negatively on the geographical and social status distances and positively on the number of common friends, with the ‘strength’ of these three factors being regulated by three parameters of the model (indicated in the sensitivity analysis below as, respectively, *h*, *r* and *k*). Once a link is created between two agents, a *weight* is assigned to the link equal to the probability of its creation, so that the interaction between pairs of agents is more ‘intense’ the closer they are geographically and socially, and the higher the number of common friends.

Because of the physiological effect of the virus (for infected agents with symptoms) and the agents’ behavioural reaction to the risks posed by the pandemic (see the previous sub-section), during the pandemic the capacity and availability of the agents to engage in social interactions may be reduced by a certain factor, which we call the agent’s *isolation rate* (*i*). The values of *i* can go from 0 to 1 for hospitalized agents. The isolation rate affects the size and the composition of the list of the agent’s daily contacts. First, the mean number of contacts for an agent is reduced by a factor equal to the product of that agent’s isolation rate and the weighted average of its contacts’ isolation rates (with the weights being the strength of the network’s links); second, the weights of the links are reduced by the contacts’ isolation rates (so that the agent is less likely to meet people with higher isolation rates).

On any given day, the probability that a given agent becomes exposed, depends on three indexes representing the *intensity* of the interaction in the three settings mentioned above, and the interactions’ *contagiousness*, which is 0 for interactions with susceptible or exposed (i.e. infected but not yet contagious) agents. Formally, an agent’s probability of becoming exposed $p_e$ is given by:

9 $$\begin{aligned} p_e = \frac{e^r-1}{e^r} \end{aligned}$$

where *r* is the *total risk of infection*, which is given by:

10 $$\begin{aligned} r = \alpha {s} + \beta {d} + \gamma {c} \end{aligned}$$

with *s* being the risk of exposure by social contact, *d* the risk of domestic exposure and *c* the risk of being infected through the social care process (multiplied by the respective proportionality factors, which are parameters of the model).

The risk of exposure by social contact, in turn, is given by:

11 $$\begin{aligned} s = n\bar{v} \end{aligned}$$

where *n* is the number of daily contacts and $\bar{v}$ is the contacts’ average contagiousness.

The risk of domestic exposure, is given by:

12 $$\begin{aligned} d = \sum _{i=1}^{h} v_i{\theta } \end{aligned}$$

where *h* is the size of the household, $v_i$ is the contagiousness of household member *i* and $\theta$ is a prudential behaviour, risk-reducing factor, which is equal to 1 if the agents are not infected or are infected but are unaware of this fact, while $\theta <1$ otherwise.

Finally the risk of exposure through social care is given by:

13 $$\begin{aligned} c = \sum _{i=1}^{k} ({{t_i{\theta })^{\phi }}}v_i \end{aligned}$$

where *k* is the number of social care interactions (the number of people met to receive or provide for care), $t_i$ is the number of hours of care in interaction *i*, $\theta$ is the test-dependent, risk-reducing factor, $\phi$ is a parameter determining how the risk grows with the time of care (in our simulations, $\phi <1$, meaning that the risk grows with time but at a decreasing speed) and $v_i$ is the contagiousness of the agent met in interaction *i*.

#### Virus infection courses

Once an agent has become exposed through one of the three spread settings described above, it is assigned one *infection course* over four possible courses, listed below in order of growing severity:

- Asymptomatic
- Mild conditions (symptomatic not hospitalized)
- Severe conditions (hospitalized not in intensive care)
- Critical conditions (in intensive care)

In accordance with empirical studies, we assume that the probabilities through which each exposed agent is assigned one of these conditions depends on the agent’s age, social status (income quintile) and gender, with the probability of developing more serious conditions growing with age, decreasing with social status and being higher for males than for females^28–34^.

Upon exposure, the agent is also assigned an *incubation period*, which, in line with empirical observations^35,36^, is drawn from a log-normal distribution with mean of about 5 days, and a *recovery period*, whose length depends on the severity level assigned to the agent (in line with the empirical findings, we assume that the more severe the infection the longer the recovery period), in order to reproduce a log-normal distribution of the recovery period with mean of about 12 days at the population level. The exposed agent is also assigned a *viral load*, $\epsilon$, which is drawn from a standard uniform distribution.

After the incubation period, the agent starts to develop symptoms (if not asymptomatic) and, in line with empirical observations^37^, we assume that the exposed agent becomes infectious 2 days before the emergence of symptoms (therefore, 3 days after exposure, on average). We assume that the agent’s contagiousness *v* is a growing function of its viral load:

14 $$\begin{aligned} v = \varepsilon ^\delta \end{aligned}$$

with $\delta$ being a parameter regulating the relationship between viral load and contagiousness.

We differentiate the conditions of symptomatic agents by assigning them a *symptoms severity index*, between 0 and 1 exclusive, with the probability of the agent being assigned a higher value increasing with its viral load and its age, decreasing with its income quintile and being higher for males than for females. The closer to 1 the symptoms severity index, the more severe are the symptoms, the greater is the reduction of the agent’s mobility and the higher the probability that the agent will take a test.

After the recovery period, some agents will die, with a probability that also depends on age, social class and gender. All other agents recover and we assume that they are immune to Covid-19 thereafter.

### The pandemic-social care interaction

The pandemic and the social care provision process affect each other, and one of our main goals in this paper is to investigate this complex relationship, especially in terms of unmet social care need inequalities across social classes.

In the previous subsection, we have already seen that the social care provision process affects the dynamics of the pandemic as the care-related interactions represent a channel through which the pandemic spreads. On the other hand, the pandemic affects both the demand and the supply of social care.

On the demand side, agents who are hospitalized receive all the care they need in hospitals, and therefore these parts of social care demand are reduced to 0 for the duration of the hospitalization period. On the other hand, we assume that infected people may develop symptoms that, although not severe enough to require hospitalization, are sufficiently debilitating that they generate additional social care needs (other than reducing the capacity to provide for social care to nil, if the agents were normally care suppliers). Further, we assume that in infected children with symptom severity above a certain threshold, though below hospitalisation level, are not able to attend school and therefore increase the child care load of their household.

On the supply side, symptomatic agents who are not hospitalized have their care supply reduced to nil if the severity of their symptoms exceeds a certain threshold or if they become aware of their infected status. If the symptom severity of non-hospitalized agents remains below the threshold, the social care they can normally supply is reduced by a certain factor which depends on their symptom severity.

Besides the pandemic’s direct effects on social care provision, the model we present is a useful tool to investigate the effects of policies implemented to prevent the spread of Covid-19, such as *lockdowns*. In the Results section below, after presenting the results for the ‘No-lockdown’ benchmark scenario, we will show the effects of two lockdown policies: a ‘full lockdown’ policy, under which the agents are not allowed to visit other households, and a ‘partial lockdown’ policy, under which people can visit other households for social care purposes. In the latter case, we expect the total care supply to increases as we assume that the normally occupied agent (i.e. workers or students) will have more time available for social care provision, given their reduced level of ‘pre-pandemic’ activity. The effect of ‘full lockdown’ case on social care supply will be more complex as, if on one hand the normally occupied agents will have more time for social care, on the other, under this policy, people are not allowed to visit other households, so there will be a decrease of carer as people in need can only count on the carers within their household. In both cases, the simulations allow us to assess the *extent* to which social care supply is affected and to take into account these effects in the assessment of the overall desirability of these policy, in comparison to other policies.

## Supplementary Information

Supplementary Information.
