# Supplementary material for: An agent-based model of social care provision during the early stages of Covid-19
Source: Sci Rep. 2022 Oct 3;12:16534. doi: 10.1038/s41598-022-20846-9 (PMC9528879; doi:10.1038/s41598-022-20846-9)
Supplement: Supplementary file 1 — Supplementary Information. [file 41598_2022_20846_MOESM1_ESM.pdf]

An agent-based model of social care provision during the early stages of Covid-19 - Supplementary Material

ABSTRACT

Social care is a frequent topic in UK policy debates, with widespread concern that the country will be unable to face the challenges posed by the increase in demand for social care. While this is a societal problem whose dynamics depends on long-term trends, such as the increase of human lifespans and the drop of birth-rates, short-term crisis, such as a pandemic, can affect the need and supply of social care to a considerable, although temporary, extent. Building on previous modelling effort of social care provision, we present an agent-based computational model to investigate social care provision in the context of a pandemic (using as an example, the early stages of the Covid-19 pandemic), and related mitigation policies, on social care demand and supply, using a proof-of-concept agent-based model (ABM). We show how policy solutions aimed at controlling the pandemic may have substantial effects on the level of unmet social care need and propose that such models may help policymakers to compare alternative containment policies taking into account their side effects on the social care provision process.

Supplementary Methods: demographic module

The model is composed of three main modules. First, the *demographic module* creates a population on the UK with a realistic demographic structure, starting in the year 1860 and running until the year 2020 in one-year time steps. Then, from the beginning of the year 2020, a *social care module* and a *Covid-19 spread module* generate, respectively, the social care provision process and the epidemiological progression of the pandemic. While the social care and Covid-19 spread modules are described at length in the main paper, we describe here the sub-modules driving the demographic dynamics.

The population is initialized with the generation of couples which are randomly distributed on a 8×12-cell grid approximating the geography of the United Kingdom. Agents live in houses which form towns, with the density of those houses varying in rough proportion to UK population density. Agents form partnerships, have children, start working (and earn an income), relocate, retire and die, according to sub-modules the details of which have been described in previous work[1–4] and a summary of which is provided below.

Agent life-course

Agents are classified as *children*, and thus care receivers, until the age of 11. At the age of 12 they become net providers of care and are classified as *teenagers*. Agents enter adulthood at the working age of 16: at this point they can either start working or continue in education, a choice that is repeated at two-year intervals, until the age of 24. These two-year intervals represent educational stages corresponding roughly to UK education levels: A-level, Higher National Diploma, Degree and Higher Degree. After education, agents become employed, taking a salary which is a function of their socioeconomic status and the education level they have reached (see the *Socioeconomic status* subsection below). When agents reach retirement age (set at 65 in these simulations), they retire from employment and begin receiving a pension which is a fixed share of their final salary (see the *Salary function* subsection below). If they retire earlier for health reasons, their pension is reduced accordingly. Mortality rates in the model follow Noble et al.[1] and use a Gompertz-Makeham mortality model until 1951. From that point we use mortality rates drawn from the Human Mortality Database[5]. Lee-Carter projections generate agent mortality rates from 2009.

Partnership Formation and Dissolution

Once they reach working age, agents can form partnerships. Agents are paired randomly with probabilities that depend inversely on the agents' geographical distance from one another, their age and socioeconomic differences. Age-specific annual divorce probabilities determine whether a couple dissolves their partnership in each year. With a certain age-specific probability the couple's female will give birth. Fertility rates are computed similarly to mortality rates: data from the Eurostat Statistics Database[6] and the Office for National Statistics[7] are used from 1950–2009, with Lee-Carter projections taking over thereafter.

Internal migration

Relocation happens most frequently due to agents finding a partner in a different town. Male agents will also relocate to new houses once a partnership dissolves, and any children produced by that partnership stay with the mother. Retired agents with care needs may move in with one of their their adult children, with a probability determined by the their care need level and the amount of care supply in their child's household. Orphaned children are adopted by a household in their kinship network, or by a random family if there are no available households in their kinship network.

Apart from these specific cases, households also relocate to another town with a certain fixed probability divided by a factor which represents the relative cost of relocation, i.e. the ratio between the total cost of relocation and the households' per capita income, with the total cost of relocation depending on the household's size and the number of years the household's member have been living in the current town. Once the household decides to relocate, another town is chosen with a probability which depends on the town's size and the town's distance from the current town. Once the town is selected, the household chooses a house among that town's empty houses, with a probability which is inversely related to the difference between the relocating household's mean social status and the average social status of the house's neighborhood.

Salary function

Every employed agent receives an hourly salary which is a function of its SES and its cumulative work experience. Formally, the salary function is the following Gompertz function (for details, see Gostoli and Silverman[3,4]). Workers start with an initial hourly wage which depends on their SES. The hourly wage increases with their work experience, at a rate which decreases with the SES, until it eventually reaches a maximum SES-specific level. In each year, the previous stock of work experience is incremented by the ratio between the agents' actual number of weekly working hours and the maximum weekly working hours (which is set to 40). Therefore, for any given SES, the hourly wage of workers working full time will grow faster than the hourly wage of workers taking hours off work.

Supplementary Discussion: social care module

Government-funded social care

As outlined in previous work[4], agents with care needs may be eligible for publicly funded care, via a government-funded care scheme based upon the framework in place in England. In this scheme, all adults with care need levels of 'critical' and whose savings are below £23,250 receive public financial support. When their savings are less than £14,250 the government pays all social care expenses which the receiver cannot afford without reducing their income below £189 per week. Above this level of savings the government subsidy is reduced by £1 for every £250 in savings. This model does not distinguish between different types of care in this scheme (i.e., at-home care or care homes, etc.).

Child Care

Here we provide a brief summary of child care processes in the simulation; we refer the interested reader to our previous work[4] for further details on the child care allocation process. In this simulation all children, with the exception of newborns, have identical childcare need, requiring 10 hours of care each day. The net care need for each child agent varies by age, given the presence of child care and education policies targeted at specific age groups. Newborns have a much higher care need which must be provided by their mother, who in turn allocates all her care supply for the newborn.

There are two significant differences in our model between social and child care, which in turn affect how we model each type of care provision. First, parents are required by law to care for their children, and therefore we assume that child care always takes priority over the provision of adult social care. This means that in our allocation process, social care supply is only allocated after child care supply is allocated. Second, child care can be delivered on a many-to-one basis, whereas social care must be delivered on a one-to-one basis.

Social care is significantly more expensive to provide than child care, with prices between three to four times higher. Within our model we assume that households will therefore prefer to allocate their *income* to provide formal child care, and their own *time* to provide informal social care, as this is the most economically viable option in most cases.

Care allocation process

In this model we adopt the care allocation process developed in our previous work[4]. We simulate care allocation as a negotiation taking place across agent kinship networks. The allocation process has two stages: 1) the available care supply (available time/income for care) is allocated to child care need; 2) remaining resources are provided to social care needs.

In both stages the allocation process randomly samples one care-receiving unit (which is an individual in the case of social care, or a household in the case of child care), with a probability proportional the unmet care need of that unit. The care receiver is then linked with a care-providing household in the receiver's kinship network; potential care-giving households are sampled with a probability proportional to that household's care supply.

After the care supplier has been chosen, a 2-hour unit of care is provided from one member of that household with available supply to the care receiver. If the supplying household is at distance I, then that household may choose to provide either time (in the form of informal care) or income (in the form of formal care).

When choosing between providing assistance in the form of income or time, the choice is made depending on the hourly wage of the worker in the supplying household with the lowest wage. If the price of formal care is higher than that wage, then the supplying agent will prefer to take time off work to provide informal care; if the price of formal care is lower, then the agent will prefer to purchase that care and remain in work.

Supplementary Discussion: pandemic-related outcomes

While the focus of our work is the effects of the pandemic on social care provision, which have been shown in Results section of the main paper, the simulations produce pandemic related outputs as well, which we show below. The next four figures show the results of simulations based on the benchmark 'no-lockdown' scenario. In Supplementary Figure S1a we can see that at the peak, just after day 60, between 1200 and 1600 people are infectious, representing approximately 15% of the population. Supplementary Figure S1b shows the number of hospitalized agents. At the peak, around 1% of the population is hospitalized.

In Supplementary Figure S2a and Supplementary Figure S2b, we begin to unveil the social gradient of health by looking at the total number of infectious and hospitalized agents by income quintiles. We can see that, in line with some empirical evidence[8, 9], the number of infectious people grows with the income quintile. In our simulations, this can be attributed to two main factors: a higher number of contacts and a higher percentage of elderly people in the higher income quintiles, compared to the lower income quintiles. However, in line with the social gradient of health, we can see from S2b that, despite higher numbers of infected individuals, agents in the the highest quintile are only half as likely to end up in hospital.

In Supplementary Figure S3a and Supplementary Figure S3b, we show the social gradients in social care provision. From Figure S3a we can see that social care need shows a clear social gradient, with the care need of the first quintile being around 20% higher than the care need of the fifth quintile. This results in the highly unequal distribution of the unmet care need we can see in Figure S3b, with the first quintile having four times the amount of unmet care need of the fifth quintile.

References

1. Noble, J. *et al.* Linked lives: the utility of an agent-based approach to modeling partnership and household formation in the context of social care. In *Proceedings of the 2012 Winter Simulation Conference (WSC)*, 1–12 (IEEE, 2012).
2. Silverman, E. E., Hilton, J. J. & Bijak, J. J. Simulating the cost of social care in an ageing population. In *Proceedings of the 27th European conference on modelling and simulation* (European Council for Modeling and Simulation, 2013).
3. Gostoli, U. & Silverman, E. Modelling social care provision in an agent-based framework with kinship networks. *Royal Soc. open science* **6**, 190029 (2019).
4. Gostoli, U. & Silverman, E. Social and child care provision in kinship networks: An agent-based model. *Plos one* **15**, e0242779 (2020).
5. Human Mortality Database 2011. https://www.mortality.org/cgi-bin/hmd/.
6. Eurostat Statistics Database. Domain Population and Social Conditions. https://ec.europa.eu/eurostat/statistics-explained/index.php/Population_and_social_conditions (2011).
7. Office for National Statistics. Birth Statistics, Series FM1 (27). https://webarchive.nationalarchives.gov.uk/20160129135406/http://www.ons.gov.uk/ons/rel/vsob1/birth-statistics--england-and-wales--series-fm1-/no--27--1998/index.html (1998).
8. Finch, W. H. & Hernández Finch, M. E. Poverty and covid-19: rates of incidence and deaths in the united states during the first 10 weeks of the pandemic. *Front. Sociol.* **5**, 47 (2020).
9. Dong, E., Du, H. & Gardner, L. An interactive web-based dashboard to track covid-19 in real time. *The Lancet infectious diseases* **20**, 533–534 (2020).

Supplementary Figure S1. Pandemic dynamics.

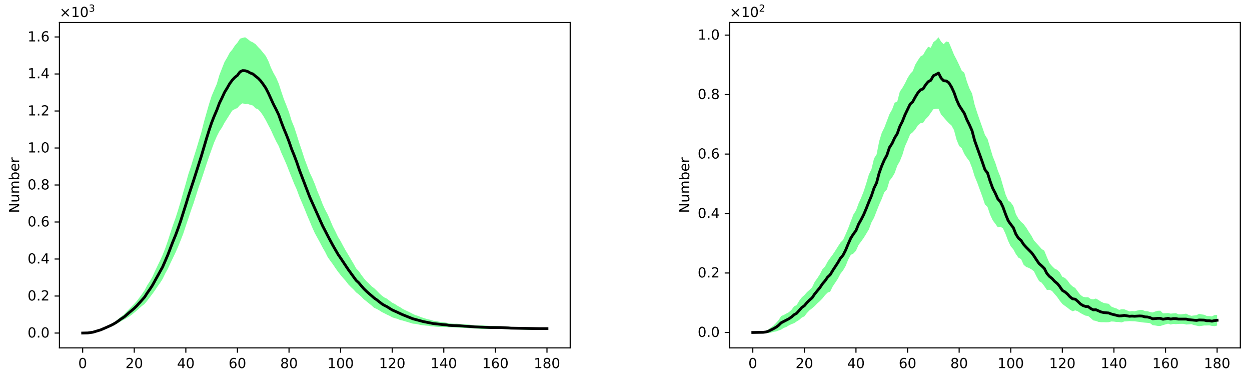

(a) Number of infectious agents.

(b) People in hospital.

Supplementary Figure S2. Social gradients of health.

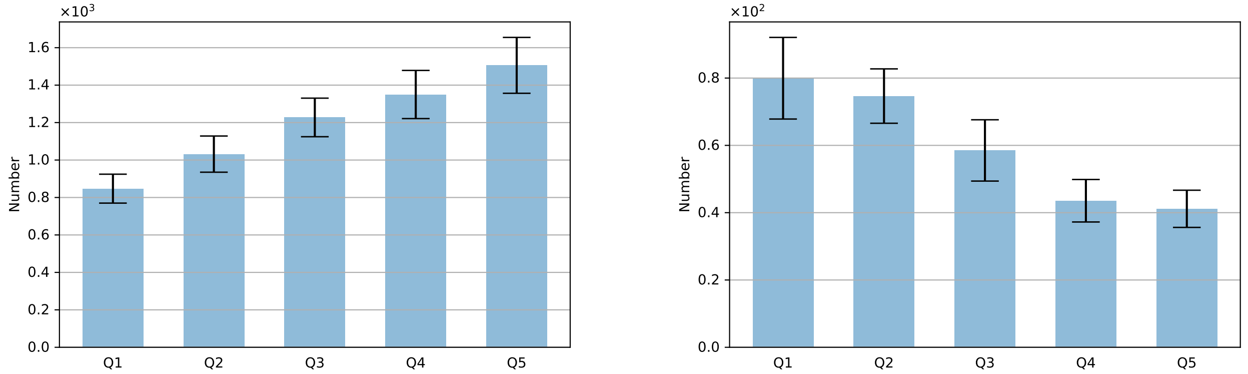

(a) Infectious by income quintile.

(b) Hospitalized by income quintile.

Supplementary Figure S3. Social gradients of care.

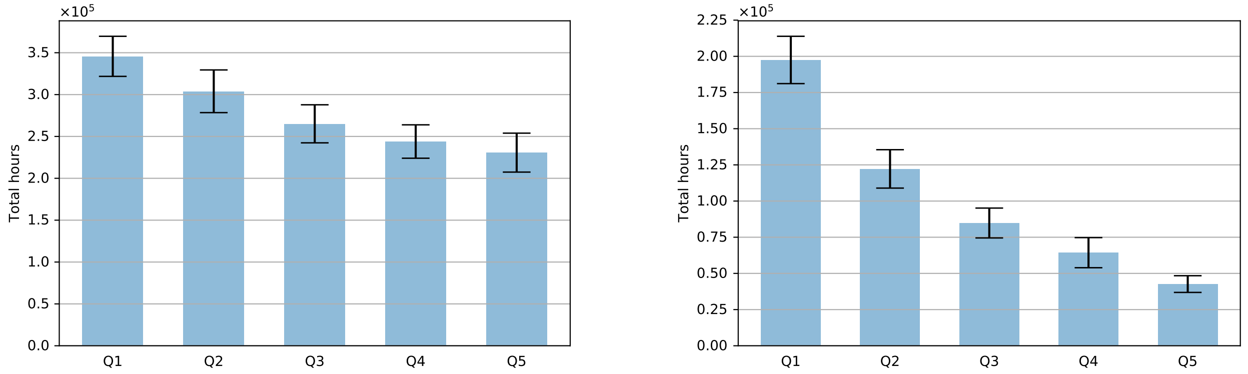

(a) Care needs by income quintile.

(b) Unmet care need by income quintile.
